# Robocasting of SiO_2_-Based Bioactive Glass Scaffolds with Porosity Gradient for Bone Regeneration and Potential Load-Bearing Applications

**DOI:** 10.3390/ma12172691

**Published:** 2019-08-22

**Authors:** Jacopo Barberi, Francesco Baino, Elisa Fiume, Gissur Orlygsson, Amy Nommeots-Nomm, Jonathan Massera, Enrica Verné

**Affiliations:** 1 Department of Applied Science and Technology (DISAT), Politecnico di Torino, 10129 Turin, Italy; 2 Interuniversity Center for the Promotion of the 3Rs Principles in Teaching and Research, 56121 Pisa, Italy; 3 Department of Mechanical and Aerospace Engineering (DIMEAS), Politecnico di Torino, 10129 Turin, Italy; 4 Department of Materials, Biotechnology and Energy, Innovation Center Iceland (ICI), 112 Reykjavik, Iceland; 5 Department of Mining and Materials Engineering, McGill University, Montreal, QC H3A OE8, Canada; 6 Faculty of Medicine and Health Technology, Tampere University, 33720 Tampere, Finland

**Keywords:** bioactive glass, additive manufacturing, 3D printing, scaffold, porosity, bioactivity, bone, tissue engineering

## Abstract

Additive manufacturing of bioactive glasses has recently attracted high interest in the field of regenerative medicine as a versatile class of fabrication methods to process bone substitute materials. In this study, melt-derived glass particles from the SiO_2_-P_2_O_5_-CaO-MgO-Na_2_O-K_2_O system were used to fabricate bioactive scaffolds with graded porosity by robocasting. A printable ink made of glass powder and Pluronic F-127 (binder) was extruded into a grid-like three-dimensional structure with bimodal porosity, i.e., the inner part of the scaffold had macropores with smaller size compared to the periphery. The crystallization behavior of the glass powder was studied by hot-stage microscopy, differential thermal analysis, and X-ray diffraction; the scaffolds were sintered at a temperature below the onset of crystallization so that amorphous structures could be obtained. Scaffold architecture was investigated by scanning electron microscopy and microtomographic analysis that allowed quantifying the microstructural parameters. In vitro tests in Kokubo’s simulated body fluid (SBF) confirmed the apatite-forming ability (i.e., bioactivity) of the scaffolds. The compressive strength was found to slightly decrease during immersion in SBF up to 4 weeks but still remained comparable to that of human cancellous bone. The pH and concentration of released ions in SBF were also measured at each time point. Taken together, these results (favorable porosity, mechanical strength, and in vitro bioactivity) show great promise for the potential application of these robocast scaffolds in bone defect repair.

## 1. Introduction

Bone defects affect a very large number of people worldwide. In the United States only, more than 6.2 million bone fractures occur every year and 10 million people are affected by osteoporosis [1]. These conditions, along with several more such as bone cancer and infection, negatively affect bone self-repairing autonomy. Bone substitute materials (BSMs) can be used in those cases to ensure the correct restoration of bone tissues; in this regard, natural or man-made materials are transplanted or implanted, respectively, during surgery by using a technique called grafting. Among the different types of graft, autografts, performed by using autogenous bone, are still considered the gold standard for bone substitution from the biological point of view but suffer from heavy drawbacks (donor site morbidity and limited availability). Allografts, on the other hand, while overcoming those issues, might lead to disease transmission and rejection of the transplanted material [2]. The alternative to these kinds of transplantation is the implantation of man-made biomaterials.

Several different BSMs were developed and artificially produced for bone grafting, i.e., biocompatible polymers, metals, ceramics/glasses, and composites [3]. Bioactive glasses (BGs) exhibit very attractive properties as materials for bone tissue engineering (BTE) thanks to their bonding ability at the implant/bone interface through precipitation of a hydroxyapatite (HA) layer [4]. Man-made materials for BTE are often produced as porous scaffolds, which should fulfill specific requirements in terms of biological features, porosity, and mechanical properties to achieve a good implant outcome [5]. Ideally, BSMs shall exhibit a hierarchical structure, with interconnected pores of different dimensions ranging from few micrometers (which promote cell adhesion) to 100–500 µm (which are key to enhance bone ingrowth and capillary vessel formation, avoiding poor vascularization) [6,7]. The total porosity is required to be at least equal to the minimum value of the trabecular bone (i.e., 50 vol.% [8]). In order to avoid stress shielding and bone resorption due to low load transfer as well as provide structural support during the whole healing process, a properly designed BSM shall show mechanical properties (i.e., compressive strength, elastic and flexural moduli) similar to those of surrounding bone [5]. Accurate control of such parameters is not achievable, unfortunately, by means of conventional fabrication techniques, which were reviewed elsewhere [7,9,10].

Solid freeform fabrication (SFF) techniques, also known as additive manufacturing (AM) processes, are very appealing for medical device manufacturing since they offer great control on the geometry and high patient-customization, which is crucial for orthopedic implants. Furthermore, supporting structures, such as columns or beams, can be embedded within the scaffold computer-aided design (CAD) model to tailor and increase the mechanical properties in the attempt to meet the very individual needs of the patient [11]. Different SFF processes have been widely studied as BSM manufacturing techniques (e.g., robocasting, stereolithography, fused deposition modeling, selective laser sintering [8]), and all of them are based on layer-by-layer construction of the scaffold starting from a computer-aided design (CAD) [12] or a text file, such as in the present work.

Robocasting is the most commonly used AM technique for the fabrication of BG scaffold, as highly porous structures (50–70 vol.%) with tailored pores ranging in size to a few hundred micrometers can be produced [13]. This process belongs to the family of direct ink-writing methods and it is based on the continuous extrusion of a filament (ink) from a printing head onto a proper substrate [14]. The ink, extruded from a fine nozzle, is a gel composed by glass powders and a polymeric binder, which needs to have pseudoplastic rheological properties, resulting in a non-flowable mass and able to sustain the overlaying scaffold layers weight during the printing process without deformation [15]. Block-copolymer Pluronic F-127 was the first binder used in 2010 for bone scaffold printing by Franco et al., who printed calcium phosphate scaffolds thanks to a hydrogel ink [16]. F-127 is currently one of the most commonly used [17,18] binder together with ethyl cellulose/poly (ethylene glycol) and carboxymethyl cellulose [19,20,21].

In the last years, numerous researches have been focused on robocasting of bioactive glasses for bone application. Fu et al., in 2011, produced BG scaffolds as strong as the cortical bone (compressive strength high as 136 MPa) by printing very fine BG powders (D_50_ = 1.2 µm) in a non-wetting oil bath, achieving a porosity (60 vol.%) comparable to that of cancellous bone [22]. BSMs for load-bearing applications must mechanically support the growing bone during all the restoration process.

Liu et al. investigated the evolution of mechanical properties both in vitro, by immersion in simulated body fluid (SBF) solution, and in vivo, using rat subcutaneous model. 13–93 glass was used to robocast grid-like scaffolds with porosity of 47 vol.% and pores as large as 300 µm. Even though the mechanical properties decreased upon 2-week in vitro and in vivo testing, scaffolds implanted in living models expressed a shift from brittle to elastoplastic mechanical behavior, showing bone-like response thanks to tissue ingrowth and supporting the opportunity for load-bearing application [19].

Commercial 45S5 Bioglass^®^, developed by Hench [23], was also used as basic material for robocasting of fully amorphous structures. In 2014, Eqtesadi et al. sintered vitreous scaffolds using a carboxymethyl cellulose-based ink. They obtained scaffolds that exhibited compressive strength (2–13 MPa) and porosity (60–80 vol.%) comparable to those of the trabecular bone [20]. The same group investigated the effect of devitrification on scaffold bioactivity in 2017 by comparing amorphous scaffolds, sintered at 550 °C, and mostly crystalline structures processed at 1000 °C. The vitreous scaffolds showed a higher conversion rate to HA due to the higher reactivity of the glassy phase upon soaking in SBF [24].

In the present paper, robocasting was used to produce fully amorphous scaffolds for bone regeneration with a grid-like structure and a porosity gradient exhibiting a denser core, in order to potentially promote rapid osseointegration in the outer shell while providing mechanical support to the host tissue. A highly bioactive SiO_2_-Na_2_O-K_2_O-MgO-CaO-P_2_O_5_ silica-based BG was used as raw material for ink preparation. This work moves a step forward in respect to a previous paper in which the same glass composition was used to fabricate robocast scaffolds with monomodal porosity, i.e., fixed pore size without any porosity gradient [25]. To the best of our knowledge, this is the first study in which robocasting was proposed to fabricate bioactive glass scaffolds with a porosity gradient.

## 2. Results and Discussion

### 2.1. Morphology and Microstructure of Robocast Scaffolds

Combined thermal analyses based on differential scanning calorimetry (DSC) and hot-stage microscopy (HSM) allowed determining the optimal sintering temperature to achieve well-densified but still totally amorphous structures. The characteristic temperatures of 47.5B glass are listed in Table 1. Glass transition (T_g_), crystallization onset (T_x_), crystallization peak (T_c_), and melting (T_m_) temperatures were obtained from DSC data, while first shrinkage (T_FS_), ball (T_B_), and flow point (T_FP_) temperatures were determined by means of HSM.

**Figure 1:**
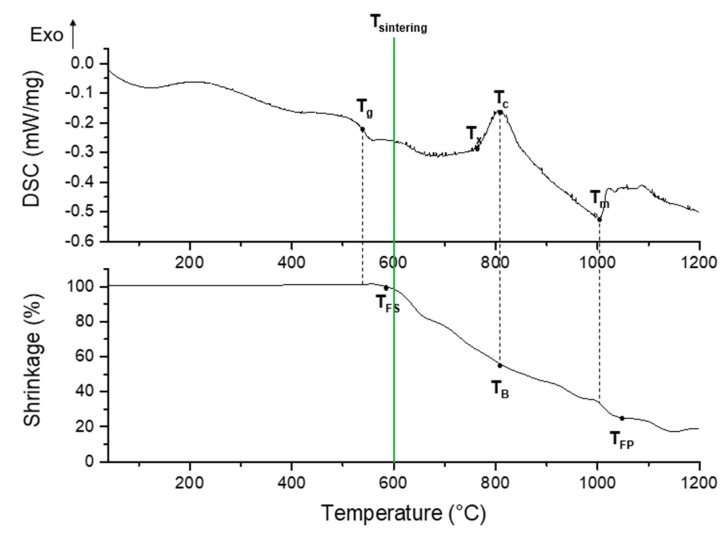
Differential scanning calorimetry (DSC) (top) and hot-stage microscopy (HSM) (bottom) plots illustrating the thermal characterization of 47.5B glass powders. Characteristic temperatures of the glass, as well as the sintering temperature chosen for scaffold fabrication, are highlighted on the plots.

The characteristic temperatures obtained from DSC analysis matched those reported by Verné et al. [26] and Fiume et al. [27], as expected. Sintering temperature was set up to 600 °C, in order to thermally treat the scaffolds just above T_FS_ but far below T_x_, thereby avoiding any crystallization phenomenon (Figure 1).

**Figure 2:**
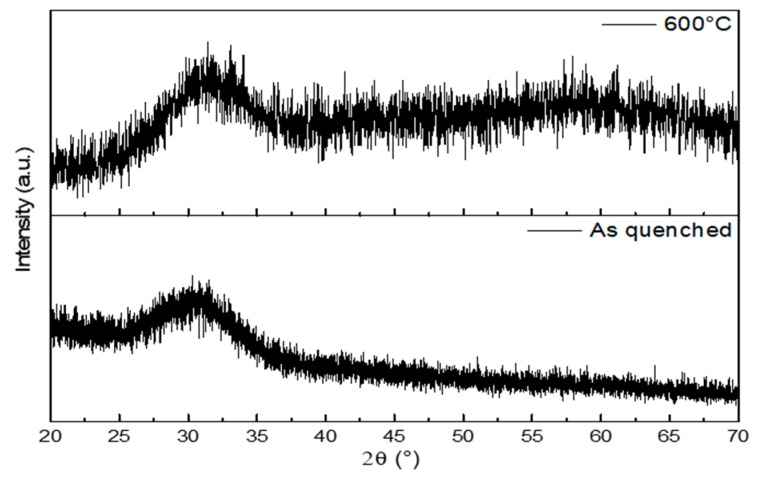
XRD patterns of as-quenched 47.5B glass powders (bottom) and ground scaffold (sintered at 600 °C for 1 h) (top). Both patterns are characterized by the glass amorphous halo.

The absence of crystalline phases was confirmed by XRD (X-ray diffraction analysis). As shown in Figure 2, the diffraction patterns of both as-quenched glass powder and sintered scaffold do not exhibit any crystalline peaks. An amorphous halo, typical of silicate glasses, was detected in the range of 2θ between 20° and 35°. These results suggest that no changes in the microstructure of the glass occurred during sintering. A fully amorphous structure is beneficial for the material bioactivity since devitrification can locally alter the dissolution characteristics of the glass and limit the formation of HA on the surface of silicate glasses due to the higher chemical stability of crystals compared to the glassy phase upon contact with biological fluids [28].

**Figure 3:**
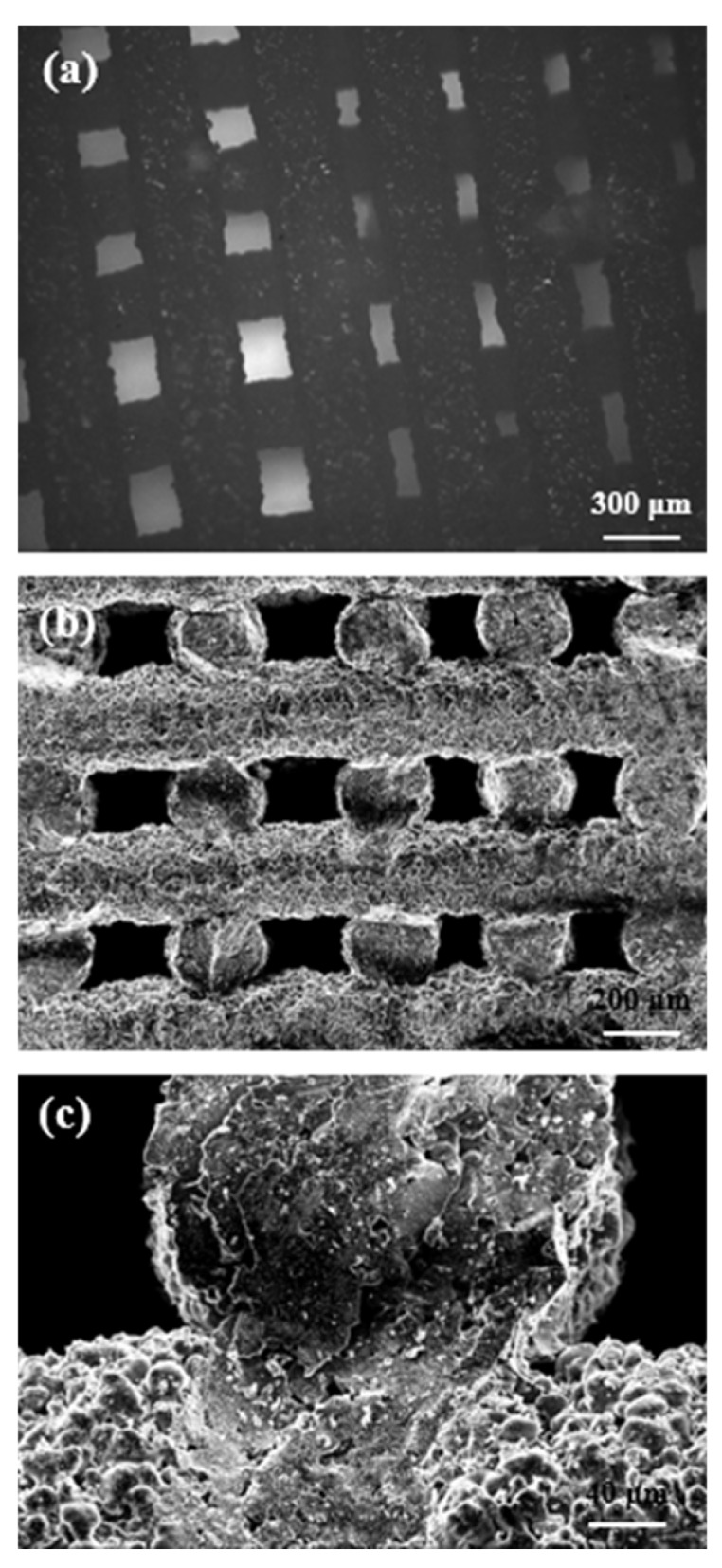
Morphological analysis of the scaffold: (**a**) top-view of the gradient of porosity and gird-like structure by optical microscopy; (**b**) SEM (scanning electron microscopy) cross-sectional image of the scaffold that was fractured to inspect the internal structure; (**c**) SEM micrograph of the trabecular section and joining region between adjacent filaments.

Morphological analyses confirmed that robocasting technique was a suitable technique to obtain highly regular structures with controlled pore-strut spacings. The scaffolds exhibit a channel-like pore structure, obtained by the orthogonal intersection of vertical and horizontal regular channels. Horizontal pores are due to spacing between layers, while vertical ones are the results of glass trabeculae distance and overlapping of tilted layers. Thus, the porosity gradient is obtained by changing the space between adjacent glass lines (Figure 3a,b). The struts have a regular circular shape and were well consolidated during the sintering process; the glass particles show a smooth surface with rounded contour, as shown in Figure 3c. The fracture plan propagating from the upper strut through the underlying layer in the same proves that the consolidation process took place between different filaments, with good fusion between layers. In this regard, a detailed description of viscous flow sintering within robocast 13–93 bioactive glass scaffolds was provided elsewhere [29].

The shrinkage due to scaffold sintering was assessed by optically measuring a large number of feature dimensions (20 measurements per each type) before and after the thermal treatment; shrinkages of 13.6%, 20.4%, and 25.2% were assessed for the large pore width, small pore width, and pore height, respectively. Shrinkage quantification potentially allows predicting the final dimensions of the product, which is very important especially if the fabrication of custom-made scaffolds is a goal.

Scaffold total porosity, evaluated by the mass-volume method, is 49.5 ± 5.5 vol.%. This value matches the minimum requirement recommended for bone substitute materials (about 50 vol.% [6]).

**Figure 4:**
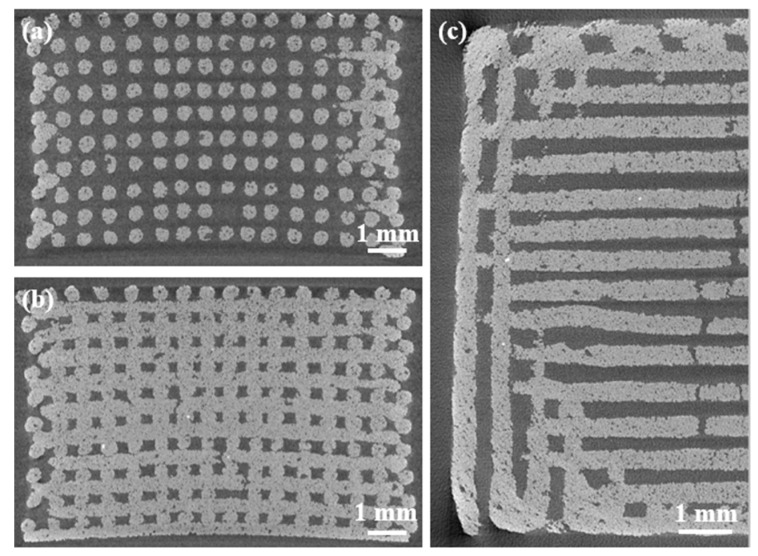
Microcomputed tomography (µCT) investigation of the scaffold structures: (**a**) cross-section on a plane passing through the gap between horizontal rods (struts); (**b**) cross-section on a plane passing through the rods; (**c**) horizontal section on a plane passing on the mid-height of the scaffold.

Further details about the pore-strut structure of scaffolds were obtained by microcomputed tomography (µCT). Bidimensional reconstructions of horizontal (x-y plane) and transversal (y-z plane) cross-sections of the scaffold are shown in Figure 4.

Tomographic images highlight the regularity of the scaffold features, such as pore dimensions, strut shapes, and size. Furthermore, the porosity gradient is well defined, in fact, the scaffolds exhibit a dense core while the porosity is larger through the peripheral layer. Defects visible in the scaffold structure are related to the fabrication process. In fact, there are both voids in the glass filaments derived from air bubbles entrapped in the ink and full-width cracks of the struts due to the different shrinkage rate of the denser core and the more porous outer shell. The slight curvature of the structure is originated by shrinkage as well.

**Figure 5:**
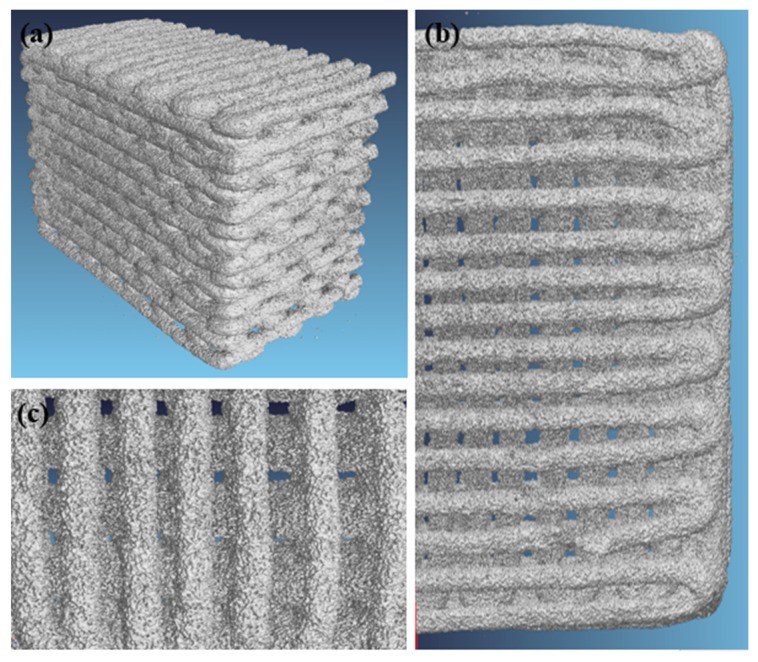
3D reconstruction of the scaffold volume by µCT analysis: (**a**) lateral view; (**b**) top view; (**c**) highlight of the change in rod spacing in the central zone of the scaffold (nominal rod diameter: 300 µm).

Cross-sectional pore and rod dimensions were measured during µCT analyses: pores height: 190 ± 30 μm; large pore width: 174 ± 18 μm; small pore width: 139 ± 32 μm; strut diameters: 275 ± 32 μm. These values are in agreement with SEM observations. Figure 5 displays a 3D volumetric reconstruction of the graded scaffold structure.

### 2.2. In Vitro Bioactivity

**Figure 6:**
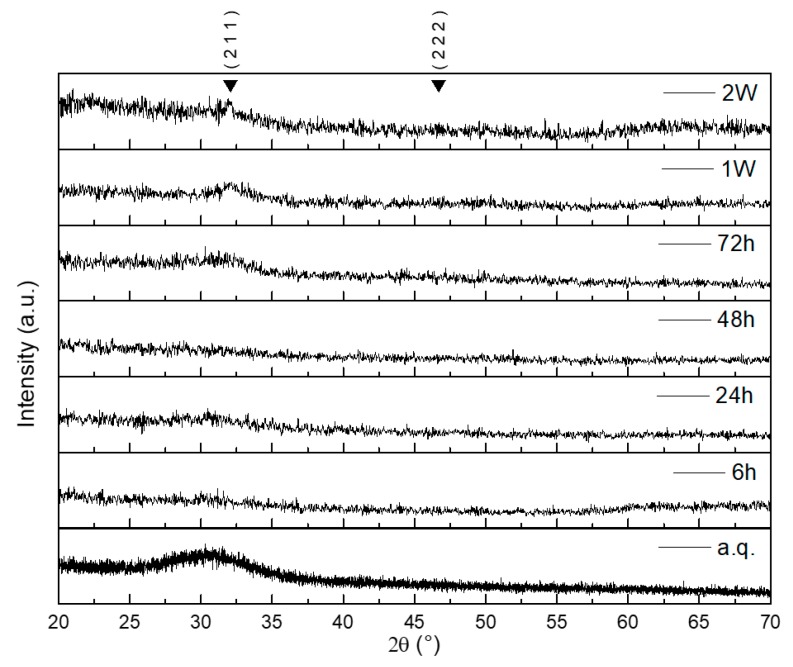
XRD patterns of graded 47.5B glass scaffolds after soaking in simulated body fluid (SBF) for different time frame. The main hydroxyapatite (HA) peaks are indicated by the Miller indices (h k l).

A glass is defined “bioactive” if it can promote the formation of HA onto its surface both in vivo (i.e., during its employment as medical device inside the human body) and in vitro (i.e., after immersion in solutions that simulate the biological environment) [30]. Formation of a surface HA layer in vitro may suggest, in spite of some limitations [31], the potential of the material to bond to bone in vivo, as the newly formed HA nanocrystals mimic the mineral phase of bone tissue and promote protein adhesion, cell attachment, and the cascade of biochemical/biomolecular events leading to osteointegration [32].

in vitro bioactivity of the scaffolds was evaluated by microstructural, chemical, and morphological analyses. XRD performed after different immersion times in SBF allowed detecting the formation of HA on the scaffold, as shown in Figure 6.

In particular, the amorphous halo in the 2θ-range between 20° and 35°, which is the typical “fingerprint” of glasses, progressively disappears over time, thus indicating the growth of a crystalline phase on the surface of the glass. Meanwhile, the major HA peak appears at 2θ = 32.2° [33]. Its broad and unsharpened appearance suggests that the newly-formed HA was constituted by nanocrystals. The second major peak of HA, referred to the (2 2 2) reflection, is just detectable at 2θ = 46.7° after immersion of the scaffold for 2 weeks in SBF. It is possible that the rough surface of the scaffold had a negative role on the XRD measurement, increasing the background and, therefore, hiding this peak.

**Figure 7:**
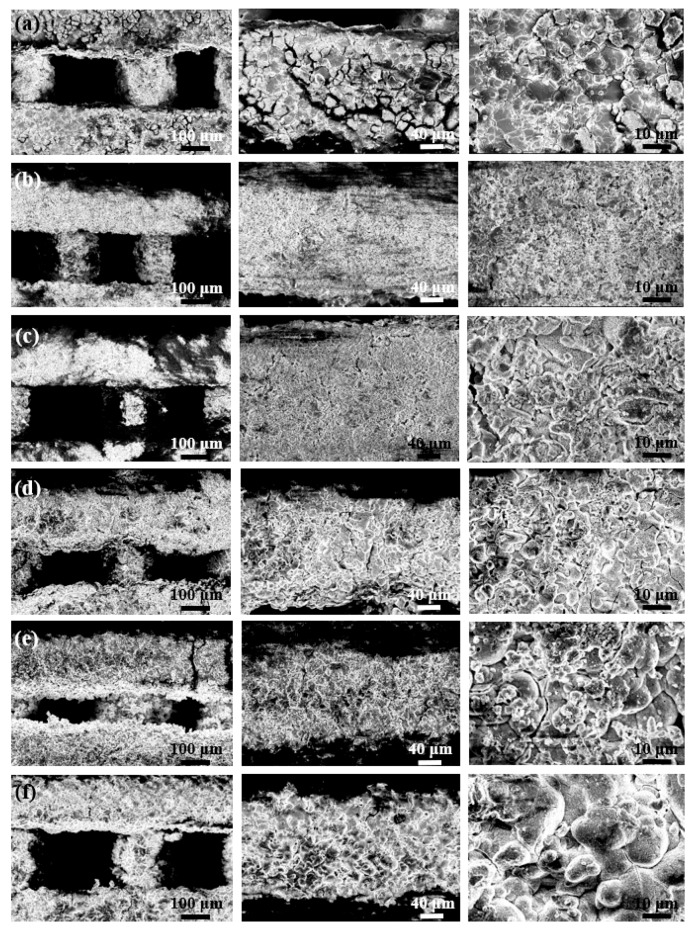
SEM topography of scaffold strut surface after different immersion times in SBF: (**a**) silica gel layer formed after 6 h soaking; (**b**) formation of HA nuclei after 24 h; (**c**) growth of acicular HA crystals during 48-h immersion; (**d**) HA layer observed after immersion time of 72 h (formed by rounded agglomerates); (**e**) rod thickening due to HA layer growth after 1 week in SBF; (**f**) after 2-week immersion a partial detachment of the HA layer was detected, revealed by almost unchanged rod diameter.

Better understanding of the bioactivity process that took place was possible thanks to morphological observation and chemical analysis by means of SEM and energy dispersive spectroscopy (EDS), assisted by µCT imaging. A five-step bioactivity process was proposed by Hench, involving: (1) ionic exchange between the glass network and the surrounding aqueous environment; (2) release of soluble silica from the glass; (3) formation of a silica gel layer on the glass surface; (4) precipitation of an amorphous CaO-P_2_O_5_ phase; and (5) crystallization of the amorphous calcium-phosphate layer into nanocrystalline HA [9]. Figure 7 shows the stepwise evolution of the scaffold surface at different SBF soaking time frame.

After 6 h, the cracked surface layer (Figure 7a) indicates the formation of the silica-gel layer. The formation of the first HA nuclei and their early growth into acicular crystals are visible after 24-h and 48-h immersion (Figure 7b,c). After longer soaking times (Figure 7d–f), the acicular crystals grew up and joined together, forming rounded agglomerates with the typical HA “cauliflower” shape, which is peculiar of bone-like apatite. After 1 week, the newly-formed HA layer is thick enough to reduce scaffold pores size due to strut thickening (Figure 7e). A detachment process of HA layer may have occurred during the second week of immersion, as suggested by the fact that rod diameter is unchanged or even reduced in some regions of the scaffold. Furthermore, the globular crystals are smaller after 2-week soaking in SBF compared to the ones forming the 1-week layer, as observed in Figure 7f. The Ca-to-P atomic ratio increases from 1.09 at 24 h to 1.33 at 1 week but remains below the theoretical value of stoichiometric HA (1.67). This is consistent with most of in vitro assessments reported in the literature on bioactive glasses that, upon being soaked in SBF, are typically coated by a layer of Ca-deficient HA [6,7].

**Figure 8:**
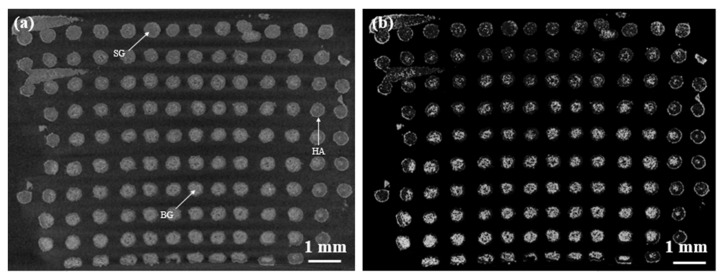
µCT cross-sections of the scaffold after 2-week SBF immersion: (**a**) standard visualization; (**b**) enhanced contrast. Different phases and reaction layers are highlighted: BG, unmodified 47.5B glass; SG, silica gel layer; HA, newly-formed hydroxyapatite.

Tomographic analysis at 2 weeks (Figure 8) reveals that the scaffold struts may exhibit differential reactivity in SBF, according to the sequence of stages of the bioactivity mechanism, depending on the spatial region where they are located. Some struts exhibit a high level of conversion to silica gel (grey tones) and a thin outer layer of HA (white tones). A second type of trabeculae, the peripheral ones, exhibit a thicker HA layer. The last kind of rods, in the core of the structure, were poorly converted into silica gel and are composed by unreacted 47.5B glass (Figure 8a). Overall, external struts seem to react with SBF at a higher rate compared to those in the core of the scaffold. Denser phases, i.e., glass and HA, are highlighted in the high-contrast image shown in Figure 8b. These differences are due to limited SBF flow and refresh in the inner part of the scaffolds during testing time, which cause differential reactivity rate—slower in the scaffold core, higher at the periphery-of the glass struts.

**Figure 9:**
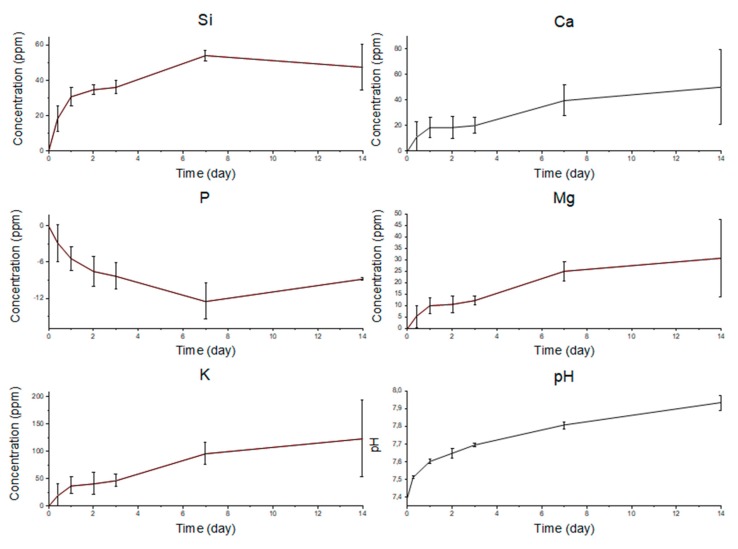
Ion concentration profile evolution during SBF soaking of Si, Ca, P, Mg, and K. The concentration variation was obtained by subtracting the ones of SBF blank. pH trend is also reported.

As suggested by other authors [34], assessing the concentration of ionic species, which are involved in the bioactivity mechanism, and the pH during soaking time in SBF is useful for in-depth evaluation of the HA formation process. Thus, the evolution of Si, Ca, P, Mg, and K ion concentrations are reported in Figure 9, alongside pH values of SBF at each time point. Even though Na^+^ ions are indeed released from the glass since the very beginning of the bioactive mechanism [35,36], the high concentration of this dissolution product within SBF solution led to oversaturation of the instrument detector; therefore, Na curve is not reported. Si concentration rapidly increased over the first week, in correspondence of the release of soluble silica Si(OH)_4_ and during the formation of the silica gel layer. Si release was limited when the HA layer started forming and its concentration became almost steady. The rapid dissolution of the glass within the first week and the decrease of the ion release rate once a thick HA layer was formed are confirmed by the concentrations of Ca, Mg, and K ions. Their trends are similar to the one of Si albeit a plateau is not reached, meaning a continuous release over the immersion time. The decrease of phosphorous ions concentration is consistent with the precipitation of HA on the scaffold struts, which still occurs at the expense of phosphate ions depletion in the solution. A similar trend was observed for other melt-derived silicate glass scaffolds under analogous in vitro testing conditions [34,35].

The pH of the solution is related to ion concentration; thus, its variation can be linked to ion exchange between the glass and the SBF. pH was measured during in vitro tests. The pH curve, shown in Figure 9, exhibits a rapid increase within the first 24 h of immersion and a lower slope during the residual time of the experiment. This is consistent with morphological observations and ion release data, indicating that a rapid release of ionic dissolution products from the glass took place over the first day, while the formation of the HA layer caused a decrease of the ion release rate.

### 2.3. Mechanical Strength

A bone substitute material, in particular for load-bearing applications, should be strong enough to support physiological loads during regeneration. Ideally, the compressive strength of a scaffold used for BTE has to match that of the trabecular bone, which falls in the range of 2–12 MPa; this value is dependent upon the age and sex of the individual [36]. Mechanical tests were performed on robocast scaffolds to evaluate if their compressive strength was suitable for load-bearing site application.

Graded robocast scaffolds exhibited a compressive strength in the range 3.8–14.4 MPa (6.1 ± 2.5 MPa), which is comparable to the range of human cancellous bone. The variability of the results derives from the brittle nature of sintered glasses and also from the manual ink mixing, which does not allow complete removal of air bubbles. The latter problem was also observed in a previous study on robocast 13–93 glass scaffolds [29]. An additional issue found with these scaffolds was elucidated by the µCT investigations (Figure 4, Figure 5); in fact, cracking within the struts due to shrinkage can have a negative and unpredictable effect on the scaffold mechanical properties. In this regard, future optimization of the process will deserve to be investigated to improve scaffold strength. Nevertheless, all the scaffolds tested were potentially suitable for load-bearing application. Moreover, the multifracture failure mechanism of the scaffolds, typical of cellular ceramics, corroborates their potential usefulness as bone substitutes even in load-bearing sites.

**Figure 10:**
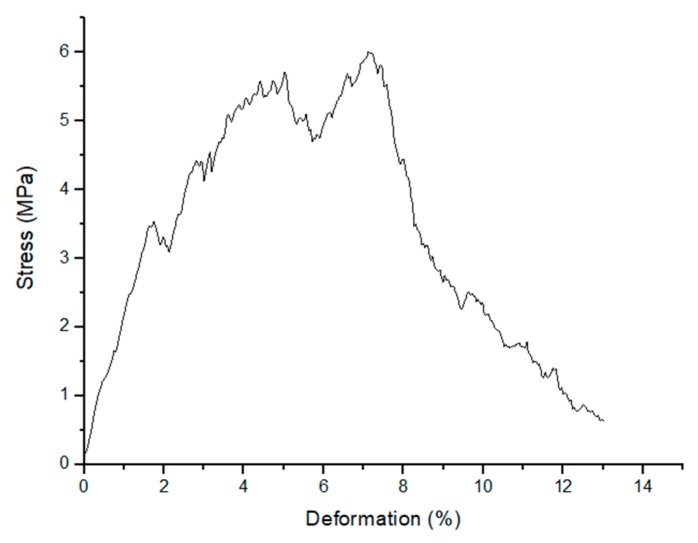
Compressive behavior (stress vs. deformation plot) of as-sintered 47.5B graded scaffolds.

As shown in Figure 10, the scaffolds can withstand several critical loads while still being able to provide adequate support to the surrounding bone. Therefore, considering an in vivo scenario, the surrounding host tissue could be mechanically supported through all the healing processes. In order to evaluate the possible decrease of mechanical strength due to prolonged contact with biological fluids, the scaffolds were immersed for two and four weeks in SBF and then tested in compression. After two weeks of immersion, compressive strength values between 3.7 and 23.5 MPa (9.9 ± 7.8 MPa) were registered. Scaffolds soaked for 4 weeks had compressive strengths in the range 2.4–23.8 MPa (10.4 ± 8.1 MPa); no statistically significant difference was found compared to the scaffolds soaked for 2 weeks in SBF. However, the compressive strength of scaffolds increased after soaking in SBF compared to untreated samples; this could be related to rod thickening due to HA formation. These results suggest that robocast 47.5B scaffolds might effectively support the regenerating bone for extended time as after prolonged soaking in SBF the compressive strength still remains comparable to that of human trabecular bone. It should also be considered that the mechanical properties of scaffolds may be different in vivo compared to those assessed in a dry state. In this regard, as observed by Liu et al. for robocast 13–93 glass scaffolds [19], the mechanical behavior of the materials once implanted in vivo may change from brittle (scaffolds in dry state or after immersion in SBF) to ductile-like due to tissue ingrowth inside pores, thus allowing higher deformations prior to failure.

## 3. Materials and Methods

### 3.1. Glass Production

As starting material for the manufacturing of the scaffolds, a silicate glass was chosen, hereafter called 47.5B (composition 47.5SiO_2_-10Na_2_O-10K_2_O-10MgO-20CaO-2.5P_2_O_5_ mol.%). It was first developed by Vernè et al. [26] at Politecnico di Torino. This glass was chosen because of its high bioactivity and large workability window (difference between the onset of crystallization (T_x_) and the glass transition temperature (T_g_), T_c_ − T_g_ = 260 °C [26]).

The glass was produced through a melting and water-casting process. A platinum crucible was filled with a homogenous mixture of the raw precursors ((SiO_2_, Na_2_CO_3_, K_2_CO_3_), (MgCO_3_)_4_·Mg(OH)_2_·5H_2_O, CaCO_3_ and Ca_3_(PO_4_)_2_ high-purity powders, Sigma-Aldrich, St. Louis, MO, USA). The reactants were heated up to 1500 °C and melted for 30 minutes. The fused glass was quenched in distilled water to obtain a frit, which was ground by using a zirconia ball miller (Pulverisette 0, Frtisch, Idar-Oberstein, Germany) and sieved below 32 μm (stainless steel sieve, Giuliani Technologies Srl, Turin, Italy; mesh 32 μm) to obtain fine particles suitable for robocasting.

### 3.2. Graded Scaffold Fabrication by Robocasting

The ink used for robocasting process was obtained by mixing glass powder and a polymeric binder (Pluronic F-127 (Sigma-Aldrich, St. Louis, MO, USA)), solution. A 27.5 wt.% optically clear solution was obtained by stirring Pluronic F-127 overnight in an ice bath, due to its thermosensitive behavior. The ink formulation was optimized through some preliminary trials and 35 vol.% of glass powder was used for the scaffold manufacturing. Glass and binder solution were mixed together into small plastic pot using a vortex mixer (Vibrofix VF1 electronic, Ika-Werke, Staufen im Breisgau, Germany) (2500 rpm) for 1 min and then cooled in ice bath for 1 min while being gently tapped to remove air bubbles as much as possible. Five mixing-cooling cycles were found to be necessary to obtain a well dispersed and homogenous glass-containing ink. Further details about these processing stages can be found elsewhere [25].

The printing plastic cartridge was loaded with the ink, which was left stabilizing at room temperature for 1 h prior to scaffold manufacturing. In this work, a pressure-control 3D printer from nScrypt was used (Tabletop-3Dn, nScrypt Inc., Orlando, FL, USA). The cartridge was connected to the printing tower, which was allowed to perform only vertical movements along the z-axis. Its position determined the printing height, with an accuracy of 5 μm. The raster pattern of the tip was determined by the horizontal movement of the metal plate under the printing tower. The precision of the plate position on the x-y plane was 10 μm [37]. In order to extrude the ink, plastic tips with inner diameter of 410 μm were used (Optimum^®^ SmoothFlow™, Nordson EFD, Westlake, OH, USA). As a printing substrate, commercially available acetate sheets (Colour Laser Printer & Copier OHP Film, Folex AG, Seewen, Switzerland) were chosen due to their flatness, the good adhesion with the ink, and the easy detachment of dried scaffolds [18].

**Figure 11:**
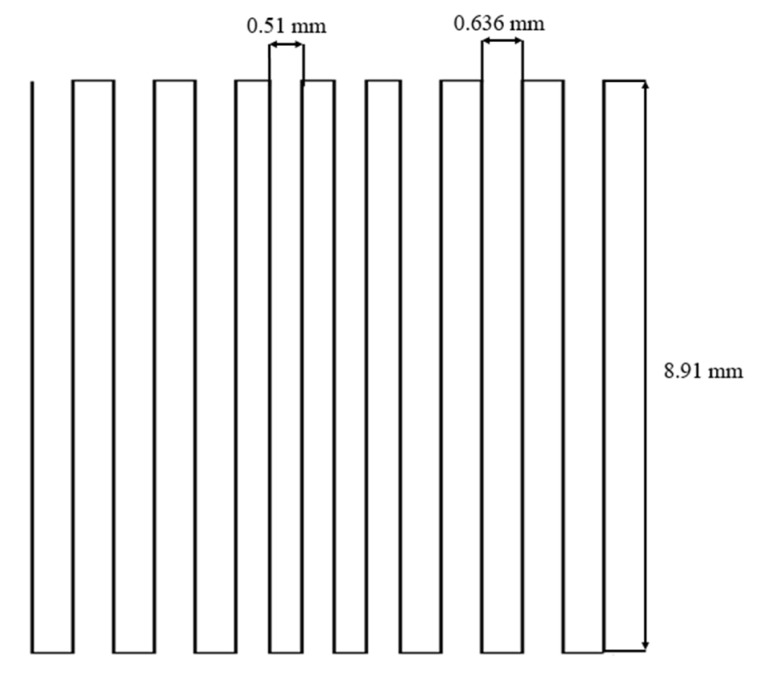
Raster pattern followed by the printing head.

Cuboid-shaped graded scaffolds were designed by superimposing 20 square layers, each one rotated of 90° with respect to the one below. The raster pattern used to build each layer is shown in Figure 11. In order to obtain the desired 3D structure, it was necessary to program every relative movement between the printing tower and the metal plate that was previously covered with an acetate sheet. Printing parameters were controlled and adjusted through the software provided by nScrypt (Machine tools 3.0): the raster speed was 2 mm/s and the extruding pressure was in the range between 1.24 and 1.51 bar.

The printing was followed by a 48-h drying of the “green” scaffolds in air at room temperature; after that, they were finally detached from the acetate sheet. The consolidation of the scaffolds was eventually obtained by a multistage thermal treatment (three steps at 200, 400, and 500 °C for 30 min each plus one at 600 °C for 1 h; heating rate 1 °C/min). The first three steps led to the burnout of the polymeric binder; it was necessary to perform them very slowly to completely remove the Pluronic F-127 and to avoid cracks related to sudden shrinkage upon heating. The actual sintering step was the last one, which was conducted above the T_g_ of the glass. At the end of the process porous graded glass scaffolds (7.0 × 7.0 × 4.5 mm^3^) were obtained.

### 3.3. Characterization

#### 3.3.1. Thermal Analysis

In order to evaluate the suitable sintering temperature for avoiding devitrification of 47.5B glass, thermal characterizations were needed, thus, differential scanning calorimetry (DSC) and hot-stage microscopy (HSM) techniques were combined. Characteristic temperatures of glass, i.e., T_g_, T_x_, T_c_, and T_m_, were obtained through DSC (STA 449 F1 Jupiter®, Netzsch, Germany) by applying a controlled thermal cycle in the range between 40 and 1200 °C (heating rate 10 °C/min) to 30 mg of glass particles in a small platinum crucible under nitrogen flowing.

The thermal behavior was also studied by mean of HSM (Expert System Solution, Modena, Italy). For this purpose, a cylinder of pressed glass powder (diameter 1 mm, height 3 mm) was obtained by using a small die and then heated to 1200 °C (heating rate of 10 °C/min) in air. One picture of the sample silhouette was taken each minute during the whole measurement in order to assess the dimensional variation and, thereby, quantify the shrinkage upon heating. Calculation and data plotting were done by using software provided by the manufacturer.

#### 3.3.2. X-ray Diffraction

Wide-angle X-ray diffraction analysis (XRD; 2θ within 20–70°) was used to investigate the possible presence of a crystalline phase in as-quenched glass powders and sintered scaffolds (properly crushed). An X’Pert Pro PW3040/60 diffractometer (PANalytical, Eindhoven, The Netherlands) equipped with Bragg-Brentano camera geometry was used for such analysis. The experimental setup parameters were: Cu Kα incident radiation (wavelength λ = 0.15405 nm); operating voltage 40 kV; filament current 30 mA; step size 0.02°; fixed counting time per step 1 s.

#### 3.3.3. In Vitro Bioactivity

The evaluation of robocast scaffold bioactivity, in terms of ionic dissolution and HA formation upon immersion in SBF, was performed by properly adapting the method developed by Macon et al. [38]. Triplicate samples of graded scaffolds were soaked in SBF, carefully prepared following Kokubo’s protocol [32], and stored at 37 °C into an orbital shaker incubator (Multitron AJ 118 g, Infors, Bottmingen, Switzerland, constant speed 100 rpm). SBF volume for each sample was calculated using a fixed value of 1.5 mg/mL for the scaffold mass/SBF volume ratio. Solution aliquots (2 mL) were withdrawn at different time points (6, 24, 48, 72, 168, and 336 h) and chemically analyzed by means of inductively coupled plasma optical emission spectroscopy (ICP-OES) (5110 ICP-OES, Agilent Technologies, Santa Clara, CA, USA) to measure the ionic release from the glass at different soaking times. HA formation was tracked at each time point by means of morphological and chemical analyses performed by field-emission scanning electron microscopy (FESEM; Supra^TM^ 40, Zeiss, Oberkochen, Germany) equipped with energy dispersive spectroscopy (EDS). Prior to FESEM inspection, the samples were sputtered with chromium. The FESEM and EDS analyses were performed using an accelerating voltage of 15 kV. XRD measurements were also performed on intact scaffolds extracted from SBF at each time point. At the end of the soaking period, scaffolds were gently rinsed with distilled water and left to dry in air at room temperature before undergoing SEM-EDS and XRD investigations.

#### 3.3.4. Morphological and Structural Characterization

The grid-like structure of scaffolds and microfeatures of the trabeculae were investigated by optical microscopy (BH2 Microscope, Olympus, Tokyo) and FESEM observation. Total porosity of the as-sintered scaffolds was calculated by mass-volume method as $\left(1-\rho /\rho_{0}\right)\times 100$, where ρ is the apparent density of the scaffold and $\rho_{0}$ is the bulk density. The measurements were carried out in quintuplicate and the porosity was expressed as mean ± standard deviation. 3D reconstructions and imaging of the scaffolds were obtained by μCT. For X-ray scanning, a Phoenix Nanotom S machine (General Electric Measurement and Control, Billerica, Massachusetts, USA; source voltage of 110 kV; source current of 110 µA; no X-ray filters) was used. The scaffolds were scanned in dry state. In order to avoid any mismatching between the 0° and the 360° shadow images, a translational motion compensation was used. Glass scaffolds were analyzed before and after 2-week soaking in SBF (scanning parameters: magnification for scaffold as such: 10.00×; after soaking: 11.11×; voxel size for scaffold as such: 5.00 μm, after 2-week soaking: 4.50 μm; rotation step: 0.50°; exposure time: 1.5 s; tube mode: 0; frame averaging number: 3; frames skipped: 1). The reconstruction of the scaffolds was obtained using the datos-x-reconstruction software, provided by the manufacturer, by means of the Radon transform [39,40]. Scaffold structure features, such as void and strut sizes, were measured by VG Studio Max 2.0 (Volume Graphics, Heidelberg, Germany). Porosity was calculated as well. BoneJ plugin [41] (version 1.4.2; ImageJ-software package, version 1.51t [42]) was employed to analyze the image stacks from VG Studio Max 2.0. Total volume, trabecular thickness, and trabecular spacing were determined through an approach used for trabecular bone investigation [41].

#### 3.3.5. Mechanical Characterization

Compression tests were performed to evaluate the mechanical behavior of the scaffolds as-such and after bioactivity evaluation (2 and 4 weeks) by using a MTS machine (Model 43, MTS Corporation, Eden Prairie, MN, USA; cell load 5 kN, cross-head speed set at 1 mm min^−1^). The resistant cross-sections of the scaffolds were measured by calipers and the failure stress was determined as the ratio between the maximum load registered during each test and the scaffold cross-section. Five samples were tested for each scaffold condition; the results were expressed as mean ± standard deviation, and the statistical differences among the groups were analyzed by Tukey’s multiple comparison test (p < 0.05).

## 4. Conclusions

This work exploits the opportunity of using robocasting to fabricate bioactive glass scaffold with a graded-porosity design for possible application as bone substitute materials also in load-bearing sites. The process setup for robocasting adopted here is relatively simple but will require future optimization for achieving better control of the sample reproducibility. Specifically, future work will be necessary to solve some issues (e.g., air-bubble entrapment in the ink, shrinkage) that can affect the quality of filaments (unwanted inner voids and cracking) and the variability of mechanical properties. However, the methodology allows good control on structural features and dimensions (pores as small as the ones in the trabecular bone were obtained). The apatite-forming ability of scaffolds upon soaking in SBF, along with the considerable mechanical strength (3.8–14.4 MPa under compression) and favorable architectural properties (total porosity 50 vol.%, large pore width 174 μm, small pore width 139 μm) support their potential suitability as bone substitute materials.

## Figures and Tables

**Table 1:** Characteristic temperatures of 47.5B glass.

Thermal Analysis Used	Characteristic Temperature	Temperature (°C)
DSC (thermal properties)	T_g_	547
	T_x_	760
	T_c_	806
	T_m_	1004
HSM (viscous behavior)	T_FS_	585
	T_B_	800
	T_FP_	1050
